# Contralateral knee osteoarthritis is a risk factor for ipsilateral knee osteoarthritis progressing: a case control study

**DOI:** 10.1186/s12891-024-07292-6

**Published:** 2024-03-02

**Authors:** Zhengxu Dai, Tao Yang, Jun Liu

**Affiliations:** 1 Department of Orthopedics, Tianjin Hospital, No. 406 Jiefang South Rd, Hexi District, Tianjin, 300211 China; 2 Department of Joints, Tianjin Hospital, No. 406 Jiefang South Rd, Hexi District, Tianjin, 300211 China

**Keywords:** Knee osteoarthritis, Side, Joint space width, Arthroplasty, Propensity score matching

## Abstract

**Background:**

Knee osteoarthritis (KOA) is a highly disabling disease, and studying its progression is crucial. However, it is still unclear whether the progression of ipsilateral knee osteoarthritis is influenced by contralateral knee osteoarthritis.

**Methods:**

Data were collected from the OAI database and divided into two study cohorts (right/left KOA cohort). Each cohort had a target knee (right/left knee) and was further divided into two groups (exposure/control group). The demographic data of both cohorts were balanced at baseline by propensity score matching (PSM), and the data included rating scale and radiographic and clinical data. After checking for balance in the matched variables, we then compared the differences between the two groups in each cohort. Our primary focus was on the minimum joint space width (mJSW) of the target knee, which was measured four years after baseline. The secondary outcome was the arthroplasty rate of the target knee within nine years.

**Results:**

In this study, a total of 678 participants were enrolled and matched. After 1:1 PSM of the baseline demographic data, 98 participants in the right KOA cohort (RKOAC) were successfully matched, and 117 participants in the left KOA cohort (LKOAC) were successfully matched. Furthermore, the standardized mean difference (SMD) of the matched variables in both cohorts was less than 0.25. After analyzing the outcome metrics, we found that the target knee had a significantly lower mJSW in the fourth year after baseline and a significantly greater arthroplasty rate within nine years in the exposed group than in the control group. RKOAC: mJSW (exposure: 2.6(1.1 ~ 3.6) vs. control: 3.3(2.0 ~ 4.2), *P* < 0.05), arthroplasty rate (exposure: 14(14.3%) vs. control: 4(4.1%), *P* < 0.05); LKOAC: mJSW (exposure: 3.1(2 ~ 3.9) vs. control: 3.4(2.6 ~ 4.2), *P* < 0.05), arthroplasty rate (exposure: 16(13.7%) vs. control: 7(6%), *P* < 0.05).

**Conclusions:**

Patients with knee osteoarthritis experienced greater progression of osteoarthritis when the contralateral knee was also affected.

## Introduction

Knee osteoarthritis is a common type of arthritis characterized by cartilage loss, joint space narrowing, osteophyte formation, synovitis, and subchondral osteosclerosis, and it can cause knee joint stiffness, pain, and limited mobility. As KOA is one of the most disabling diseases, it is estimated that more than 10% of people older than 60 years are affected by this disease. Pain, disability pensions and arthroplasty have caused significant burdens on individuals and society [1–3]. Despite the increasing sophistication in the treatment and management of KOA, there are still patients suffering from pain and a low quality of life. This is why it is important to determine what factors influence the occurrence and development of KOA.

Many factors can influence the progression of KOA to varying degrees. These include age-related cartilage degeneration, sex-related metabolism and regulatory differences, genetic and racial differences in cartilage formation, body mass index (BMI)-related cartilage wear, and other factors, such as medical interventions, comorbidities, and differences in income, diet, and lifestyle [4–10]. As mentioned above, the progression of KOA is closely associated with cartilage loss and is characterized by clinical signs and symptoms such as decreased mJSW score on imaging, pain, and limited daily living [3].

To better treat and manage KOA, much research has been conducted. Moreover, Campbell showed that ipsilateral knee flexion contracture is associated with worsening of contralateral knee function and contracture [11], and Metcalfe indicated that 80% of unilateral KOA cases develop into bilateral KOA within 12 years [12]. These studies indicate that one knee may be affected by osteoarthritis in the contralateral knee and progress to osteoarthritis. However, when one knee already has osteoarthritis, it is not yet clear whether its progression is affected by contralateral KOA. Because bilateral KOA (5%) is more common than unilateral KOA (2%) in the clinic [13], it is essential to determine whether the progression of KOA is influenced by contralateral knees with osteoarthritis. In our study, we evaluated the progression of knee osteoarthritis by mJSW and the future arthroplasty rate because they reflect the amount of cartilage and the severity of osteoarthritis, respectively.

## Method

### Data collection

Our data were collected from a public database called The Osteoarthritis Initiative (OAI, https://nda.nih.gov/oai/full_downloads.html), The OAI is a multicenter, ten-year observational study of men and women sponsored by the National Institutes of Health that involved 4796 participants. The goals of the OAI are to provide resources to enable a better understanding of the prevention and treatment of knee osteoarthritis, one of the most common causes of disability in adults. In our study, we followed Kellgren and Lawrence’s recommendation (Grade 0, normal; Grade 1, suspicious narrowing of the joint space; Grade 2, narrowing of the joint space with traces of osteophyte production; Grade 3, moderate narrowing of the joint space with sclerotic changes in the cartilage; Grade 4, narrowing of the joint space with amounts of osteophytes and cartilage deformities) that a Kellgren–Lawrence grade (KLG) ≥ 2 indicates knee osteoarthritis [14]. The inclusion criterion was a KLG grade ≥ 2 in at least one knee at baseline. The main exclusion criteria are as follows: (1) arthroplasty was found in both knees at baseline or within 9 years of study. (2) Patients whose lower extremities were subjected to other types of trauma or surgery, such as bone fracture and internal fixation surgery for fracture. (3) Data missing from KLG. (4) Patients with rheumatoid arthritis, psoriatic arthritis, or other inflammatory arthritis.

### Variables

Based on published studies, variables that have an impact on KOA were included. The variables included age (45–79 years), sex, race (yellow, white, black, others), and income (annual income; the dividing line is 50 thousand dollars). BMI (subjects were asked to first remove their heavy clothing, hats, socks, and shoes and then begin to measure and calculate). The Charlson comorbidity index (CCI) was used to quantify the type and severity of disease. Treatment consisted of medical treatment (oral drug or intraarticular injection medicine) [15, 16]. Short form-12 (SF-12, a health-related quality of life questionnaire). The Western Ontario and McMaster Universities Osteoarthritis Index (WOMAC) was used to evaluate the patients’ degree of pain, stiffness, and sensory difficulty. KLG (radiographic KLG) readings were performed by experienced musculoskeletal radiologists and rheumatologists from Boston University [17]. mJSW (minimum joint space width read from the X-ray of the knee; the unit is millimeters). All enrollment data were collected at baseline, and primary and secondary outcomes were measured in the fourth and ninth years of follow-up, respectively. In addition, the clinical and imaging data of the OAI study were obtained with the permission of four clinical centres and the local ethics committee, and all participants signed informed consent forms.

### Study design

**Fig. 1:**
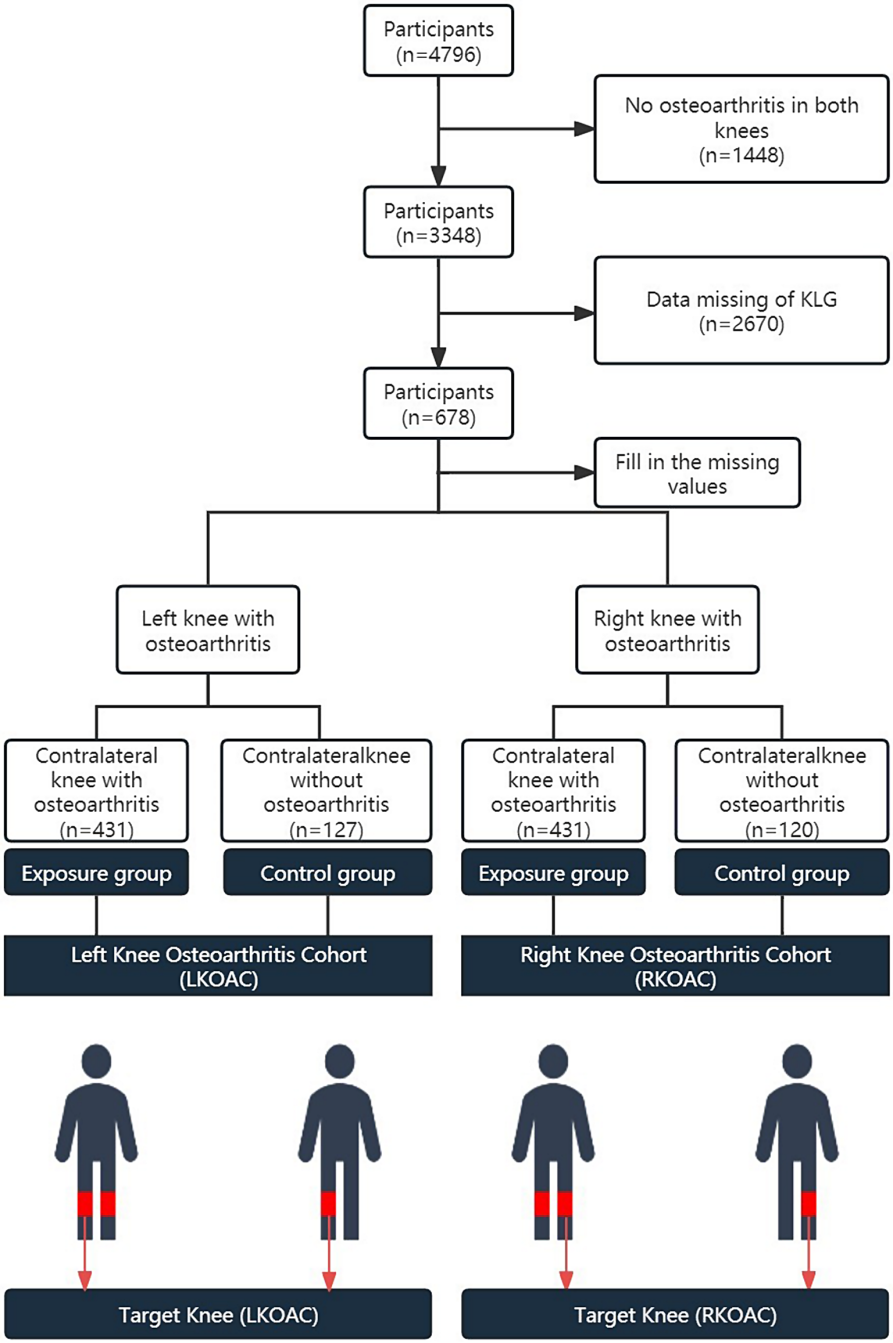
Flow diagram of participants and study design about this research

After collecting data according to the inclusion and exclusion criteria, we obtained two types of data: bilateral KOA and unilateral KOA patients. As randomly selecting the target knee would ignore differences in the knee and bias the results, we conducted the study separately for each bilateral knee. Therefore, we divided all participants into two cohorts—RKOAC and LKOAC. In RKOAC, the target knee was the right knee, and in LKOAC, the target knee was the left knee. Both groups had osteoarthritis in their target knee. The difference between the exposure group and the control group was that the exposure group’s contralateral knee also had osteoarthritis, while the control group’s contralateral knee did not have osteoarthritis. Moreover, to ensure more precise results and conclusions, we aimed for the only difference between the exposed and control groups to be the presence or absence of contralateral KOA. This required us to minimize the differences in the subjects’ conditions and the target knee itself. Therefore, we utilized PSM, a data management method commonly used in observational studies, to reduce variable bias and balance the variables between the two groups in each cohort [18]. Figure 1 shows the specific cohort information. Finally, we strictly followed the inclusion and exclusion criteria to acquire data from the OAI database.

### Statistical analysis

After identifying outliers and filling in missing values (filled with mean value) [19], well-managed data were processed via PSM. First, we selected a binary logistic regression model to calculate the propensity score (PS). Second, the nearest and did not put back matching method was adopted between the two groups according to the PS we obtained before; in addition, the maximum calliper width of matching was 0.02, and the matching ratio was 1:1 [20]. Age, sex, race, BMI, CCI, medicine, income, SF-12 score, WOMAC score, KLG, and mJSW score of the target knee were matched variables, and the presence or absence (1,0) of contralateral KOA was the grouping variable. The SMD is used to evaluate the balance of variables, and an absolute value less than 0.25 means that the variables are well balanced after matching [21, 22]. We subsequently analysed the participants after matching, and the primary outcome was mJSW in the fourth year after baseline, while the secondary outcome was the incidence of knee arthroplasty (KA) after nine years of follow-up. After checking for normality, continuous nonnormal data were expressed as medians and upper and lower quartiles, categorical data were expressed as quantities and proportions, and the Mann‒Whitney U test was used for comparisons between groups. SPSS 22.0 and R2.15 were used for data management, and a P value < 0.05 was considered to indicate statistical significance.

## Results

### Before PSM

After the inclusion and exclusion criteria were met and missing data were filling (missing value filling information: Age:3/0.4%, BMI:3/0.4%, SF-12:12/1.7%, Income:25/3.7%, CCI:21/3.1%, mJSW: 8/1.2%), a total of 678 participants were enrolled at baseline. There were 431 cells in the exposure group and 120 in the control group (Table 1) and 431 in the exposure group and 127 in the control group (Table 2). The Mann‒Whitney U test showed that there were significant differences between the two groups of variables, except for age, sex, SF-12 score, income, medicine, and CCI in the RKOAC cohort and age, sex, SF-12 score, income, BMI, and CCI in the LKOAC cohort. It is necessary to minimize the possible effects of differences between the two groups on KOA incidence. Therefore, after the above processing, we input the processed data into the PSM.

**Table 1:** Demographic data of RKOAC in baseline

	Before PSM					After PSM			
Variables	Control		Exposure	Z/P		Control		Exposure	Z/P
	n = 120		n = 431			n = 98		n = 98	
**Age**	61(53 ~ 70)		63(57 ~ 70)	0.1/0.18		63(54 ~ 70)		62(56 ~ 69)	0.5/0.6
**BMI**	28.6(26.0 ~ 31.6)		30.3(27.2 ~ 33.9)	3.3/<0.05		29.5(26.6 ~ 32.4)		28.9(26.3 ~ 31.6)	0.7/0.51
**RWOMAC**	3(0 ~ 13.9)		11.5(2 ~ 25)	5.1/<0.05		6.1(0 ~ 17.3)		7(2 ~ 15.3)	0.5/0.63
**RJSW**	4(3.0 ~ 4.9)		3.7(2.6 ~ 4.7)	2.1/<0.05		3.8(2.7 ~ 4.6)		3.7(2.5 ~ 5.1)	0.5/0.6
**SF-12**	41(39 ~ 42)		41(39 ~ 43)	0.05/0.09		41(38 ~ 42.2)		41.5(39 ~ 43)	1.5/0.14
**Sex**				0.8/0.42					0.4/0.67
male	59(49.2%)		194(45%)			50(51%)		53(54.1%)	
female	61(50.8%)		237(55%)			48(49%)		45(45.9%)	
**Race**				3.4/<0.05					0.4/0.69
others	2(1.7%)		6(1.4%)			1(1%)		0	
white	105(87.5%)		314(72.8%)			84(85.7%)		84(85.7%)	
black	13(10.8%)		109(25.3%)			13(13.3%)		13(13.3%)	
yellow	0		2(0.5%)			0		1(1%)	
**CCI**				1.1/0.27					0.5/0.63
0	92(76.7%)		305(70.8%)			75(76.5%)		72(73.5%)	
1	17(14.1%)		84(19.5%)			14(14.3%)		14(14.3%)	
2	5(4.2%)		34(7.9%)			4(4.1%)		10(10.2%)	
3	3(2.5%)		5(1.2%)			3(3.1%)		2(2%)	
> 3	3(2.5%)		3(0.7%)			2(2%)		0	
**Income(5 W)**				1.8/0.07					0.8/0.46
Y	78(65%)		241(55.9%)			61(62.2%)		66(67.3%)	
N	42(35%)		190(44.1%)			37(37.8%)		32(32.7%)	
**R-KLG**				1.9/0.06					0.5/0.62
2	75(62.5%)		218(50.6%)			55(56.1%)		50(51%)	
3	34(28.3%)		185(42.9%)			33(33.7%)		40(40.8%)	
4	11(9.2%)		28(6.5%)			10(10.2%)		8(8.2%)	
**Medicine**				0.7/0.48					0.4/0.67
Y	67(55.8%)		225(52.2%)			51(52%)		48(49%)	
N	53(44.2%)		206(47.8%)			47(48%)		50(51%)	

There is no significant difference of all variables between two groups after PSM

**Table 2:** Demographic data of LKOAC in baseline

	Before PSM					After PSM			
Variables	Control		Exposure	Z/P		Control		Exposure	Z/P
	n = 127		n = 431			n = 117		n = 117	
**Age**	62(56 ~ 70)		63(57 ~ 70)	0.2/0.83		61(55 ~ 70)		62(54 ~ 70)	0.2/0.87
**BMI**	29.3(26.4 ~ 32.7)		30.3(27.2 ~ 33.9)	1.8/0.07		29.3(26.4 ~ 32.7)		28.9(26.4 ~ 32.2)	0.4/0.65
**LWOMAC**	5(0 ~ 19.8)		10(1 ~ 28)	2.1/<0.05		5(0 ~ 23.5)		9(1 ~ 24)	1.1/0.26
**LJSW**	4(3.2 ~ 4.7)		3.9(2.9 ~ 4.8)	0.8 < 0.05		3.9(3.1 ~ 4.7)		3.9(2.9 ~ 4.9)	0.3/0.76
**SF-12**	41(39 ~ 43)		41(39 ~ 43)	1.1/0.27		41(39 ~ 43)		41(39 ~ 42)	1.1/0.27
**Sex**				1.4/0.15					0.3/0.79
male	48(37.8%)		194(45%)			46(39.3%)		44(37.6%)	
female	79(62.2%)		237(55%)			71(60.7%)		73(62.4%)	
**Race**				3.0/<0.05					0.4/0.68
others	0		6(1.4%)			0		1(0.9%)	
white	112(88.2%)		314(72.8%)			102(87.2%)		98(83.7%)	
black	15(11.8%)		109(25.3%)			15(12.8%)		17(14.5%)	
yellow	0		2(0.5%)			0		1(0.9%)	
**CCI**				0.7/0.49					1.1/0.27
0	94(74%)		305(70.8%)			87(74.3%)		79(67.5%)	
1	22(17.3%)		84(19.5%)			20(17.1%)		26(22.2%)	
2	8(6.3%)		34(7.9%)			8(6.8%)		10(8.5%)	
3	1(0.8%)		5(1.2%)			1(0.9%)		1(0.9%)	
> 3	2(1.6%)		3(0.7%)			1(0.9%)		1(0.9%)	
**Income(5 W)**				0.3/0.76					0.7/0.51
Y	73(57.5%)		241(55.9%)			69(59%)		64(54.7%)	
N	54(42.5%)		190(44.1%)			48(41%)		53(45.3%)	
**L-KLG**				2.0/<0.05					0.4/0.69
2	85(66.9%)		245(56.8%)			75(64.1%)		73(62.4%)	
3	35(27.6%)		154(35.7%)			35(29.9%)		34(29.1%)	
4	7(5.5%)		32(7.4%)			7(6%)		10(8.5%)	
**Medicine**				2.2/<0.05					0.5/0.6
Y	52(40.9%)		225(52.2%)			51(43.6%)		47(40.2%)	
N	75(59.1%)		206(47.8%)			66(56.4%)		70(59.8%)	

There is no significant difference of all variables between two groups after PSM

### After PSM

**Fig. 2:**
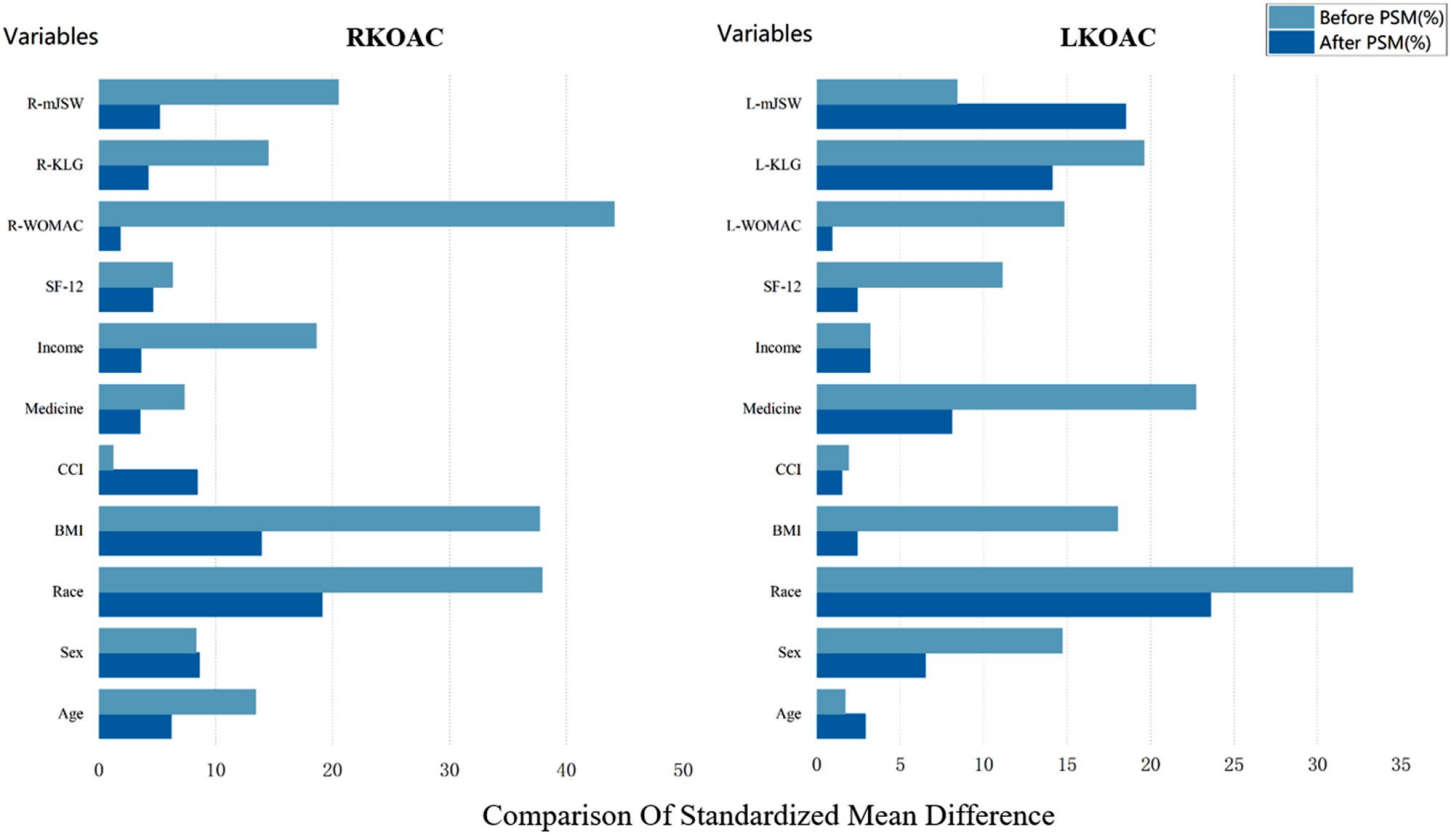
Comparison of SMD of R/LKOAC. SMD of all variables are lower than 25% after PSM, it means balanced well

A total of 98 RKOAC (Table 1) and 117 LKOAC (Table 2) cells were successfully matched after PSM of the baseline processed data. Upon conducting a balance test, it was observed that the majority of variables in both cohorts displayed lower SMD values after matching. While a handful of variables (sex, CCI in RKOAC cohort and age, income, LJSW in LKOAC cohort) exhibited higher SMD values, all the variables displayed absolute SMD values less than 0.25 after matching. This indicates that a commendable level of balance was achieved (Fig. 2). Moreover, the Mann‒Whitney U test revealed no statistically significant differences in variables between the two groups in either cohort (Tables 1 and 2). Consequently, a subsequent matched data study could be conducted.

#### Outcomes

We calculated and compared the mJSW score at the fourth year after baseline and the KA rate after nine years of follow-up about the target knee. We found that the target knee had a significantly lower mJSW in the fourth year after baseline and a greater arthroplasty rate within nine years in the exposure group than in the control group. The results for two outcome indicators in each cohort are shown below:

Primary outcome: RKOAC: mJSW (exposure: 2.6 (1.1 ~ 3.6) vs. control: 3.3 (2.0 ~ 4.2), *P* < 0.05); LKOAC: mJSW (exposure: 3.1 (2 ~ 3.9) vs. control: 3.4 (2.6 ~ 4.2), *P* < 0.05) (Table 3).

**Table 3:** Results of R/LKOAC

**RKOAC**	Primary outcome	mJSW (Median (upper-lower quartile))	Exposure	Control	Z/P-value
		Baseline	3.7(2.5 ~ 5.1)	3.8(2.7 ~ 4.6)	0.5/0.6
		Four years	2.6(1.1 ~ 3.6)	3.3(2.0 ~ 4.2)	2.9/<0.05
	Secondary outcome	KA rate (baseline-9 years)	98/14(14.3%)	98/4(4.1%)	2.5/<0.05
**LKOAC**	Primary outcome	mJSW (Median (upper-lower quartile))	Exposure	Control	Z/P-value
		Baseline	3.9(2.9 ~ 4.9)	3.9(3.1 ~ 4.7)	0.3/0.76
		Four years	3.1(2 ~ 3.9)	3.4(2.6 ~ 4.2)	2.3/<0.05
	Secondary outcome	KA rate (baseline-9 years)	117/16(13.7%)	117/7(6%)	2.0/<0.05

mJSW was balanced at baseline and no significant difference between two groups. After four years, two groups’ mJSW are different and exposure group is lower than control group, the P-value less than 0.05. It means exposure group has higher articular cartilage loss. Besides, higher KA rate also indicates that exposure group’s participants may have more severe progression of KOA.

Secondary outcomes: RKOAC: arthroplasty rate (14 (14.3%) vs. control: 4 (4.1%), *P* < 0.05); LKOAC: arthroplasty rate (16 (13.7%) vs. control: 7 (6%), *P* < 0.05) (Table 3).

## Discussion

The articular cartilage of the knee is a thin layer of special connective tissue with good viscosity and elasticity that can significantly decrease the friction coefficient of the articular surface of the knee [23]. In addition, special material properties enable the cartilage to withstand high contact forces. Moreover, it also forms a dynamic bearing structure with the subchondral bone and disperses the force it receives to the subchondral bone to protect the knee well [24]. Therefore, it is difficult to ignore the role of articular cartilage in studies on KOA. Giuseppe Musumeci et al. indicated that, whether the damage is caused by inflammatory and metabolic factors or mechanical wear, the target of damage in KOA patients is articular cartilage [25], and an important index for evaluating cartilage loss is the joint space on imaging [3]. In our study, through a strict research design, we found that knees with osteoarthritis have a greater mJSW decrease and greater incidence of future KA when the contralateral knee also has osteoarthritis. Similarly, Wirth et al. included one hundred twenty participants and followed up for four years and reported a greater quantity of articular cartilage loss in a knee with osteoarthritis when the contralateral knee suffered from osteoarthritis by evaluating cartilage thickness changes on MRI images of the knee [26]. Eckstein F et al. also reported that the speed of articular cartilage loss is quicker when the contralateral knee has severe osteoarthritis than when the contralateral knee has earlier osteoarthritis through one year and four years of follow-up of one hundred fifty participants [27]. A greater degree of articular cartilage loss indicates more severe and frequent pain and activity difficulty, and the failure of two knees causes a vicious cycle, leading to a worse outcome [28].

Differ from previous studies which don’t differentiate the side of the knee or just select a more severe or less severe side to study, and conclude a normal knee is easier affected and becomes worse when the contralateral knee has osteoarthritis [12, 29, 30]. Our study divided right and left knees into two cohorts, and each cohort was divided into two groups, making this research more rigorous than them. There are also some papers that have evaluated the severity of KOA through cartilage change, but they did not balance the variables in baseline demographics that have an effect on the target knee, making the conclusion less convincing [31–34]. In our study, PSM was used to balance all covariates according to the baseline demographics of the two groups. Factors that can influence the development of KOA were not significantly different between the two groups. The only difference between the two groups was whether the patient had osteoarthritis in the contralateral knee. This could greatly reduce the interference of confounding factors. In the data selection, we strictly followed the inclusion and exclusion criteria and deleted data that did not meet the criteria. The covariates we selected have already been confirmed to be the main risk factors affecting the development of KOA through published studies.

There are also several limitations in our study. First, although the use of PSM to balance covariates among baseline demographic variables has made our conclusions more convincing, it also brings limitations. Here may still be some variables that have an impact on the development of KOA that we have not included, such as a history of lower limb trauma and the use of sodium hyaluronate treatment through intra-articular injection [35, 36]. This means that we did not balance these variables, and they may influence the accuracy of the conclusions we obtained [37]. Second, due to limitations in terms of the data and participants involved in the OAI, we considered only the variables mentioned above and variables such as the CCI and medicine, including many additional subdivided items such as heart disease and diabetes in the CCI, nonsteroidal anti-inflammatory drugs (NSAIDs), and salicylic acid in medicine. The follow-up date was also limited to two or four years because of the fixed follow-up date. In addition to variable selection, uncontrollable factors, such as changes in gait pattern, load force of the knee joint, and decreases in physical activity, cannot be ignored. These changes can lead to adaptive changes in the hip and ankle and further impact the ipsilateral and contralateral knee through joint interaction with the lower limbs [38–40], these changes may also influence the accuracy of our results and conclusions. Finally, a retrospective study has several limitations itself, the most important point is that our conclusions cannot be applied to the clinical diagnosis and treatment of KOA directly in patients aged less than forty-five years and more than seventy-nine years. However, additional support from similar studies is needed, especially for prospective randomized controlled trials. Nevertheless, our study clarified the relationship between the progression of KOA on one side and contralateral KOA.

## Conclusions

Knee with osteoarthritis had lower minimum joint space width and higher future arthroplasty rate when the contralateral knee was also had osteoarthritis compared with not. These findings will be of great help in the management and treatment of patients with bilateral KOA, which is more common in clinical practice. Therefore, we recommend paying more attention to patients who have osteoarthritis in both knees. This includes close monitoring and regular follow-up to observe any changes in the disease. Early intervention on one knee with osteoarthritis, such as rehabilitation, functional exercise, drugs, or surgical treatment, if indicated, can delay the development of osteoarthritis in the other knee. These methods have significant clinical value in terms of pain relief and improvement in quality of life.

## Data Availability

All data can be found in the OAI database: https://nda.nih.gov/oai/full_downloads.html. Upon successful registration and login, utilize the link URL above to gain access to all data.

## Abbreviations

KOA: knee osteoarthritis
PSM: propensity score matching
RKOAC: right KOA cohort
LKOAC: left KOA cohort
mJSW: minimum joint space width
SMD: standardized mean difference
KLG: Kellgren-Lawrence grade
PS: propensity score
BMI: body mass index
CCI: Charlson comorbidity index
SF-12: Short form − 12
KA: knee arthroplasty
OAI: The Osteoarthritis Initiative
NSAIDs: nonsteroidal anti-inflammatory drugs
WOMAC: The Western Ontario and McMaster Universities Osteoarthritis Index
